# Case Report: Intravascular large B-cell lymphoma presenting with cauda equina syndrome: a rare case and diagnostic dilemma

**DOI:** 10.3389/fmed.2026.1873276

**Published:** 2026-08-05

**Authors:** Xu-yang Zhang, Han-sheng Fang, Xin-yue Liu, Hai-dan Chen, Hui-li Cai

**Affiliations:** 1 Department of Hematology, The First College of Clinical Medical Science, China Three Gorges University, Yichang, Hubei, China; 2 Department of Spinal Surgery, The First College of Clinical Medical Science, China Three Gorges University, Yichang, Hubei, China

**Keywords:** cauda equina syndrome, diagnostic delay, interleukin-10, intravascular large B-cell lymphoma, random skin biopsy

## Abstract

**Objective:**

To investigate the clinical and diagnostic characteristics of intravascular large B-cell lymphoma (IVLBCL) presenting primarily as cauda equina syndrome (CES), analyze factors contributing to diagnostic delay, and evaluate the diagnostic value of minimally invasive biopsy.

**Methods:**

We report a case of IVLBCL that initially presented with CES. The diagnostic process was reviewed by analyzing serum biomarkers and pathology from a random skin biopsy (RSB) that included subcutaneous fat tissue. Additionally, a literature review was conducted via the PubMed database (2005–2025), which identified and analyzed 10 additional cases of IVLBCL with CES as the initial manifestation. Imaging features, clinical symptoms, and time-to-diagnosis were synthesized across these cases.

**Results:**

In the present case, the patient presented with progressive lower extremity weakness, saddle anesthesia, and sphincter dysfunction, while early spinal MRI findings were unrevealing. Diagnostically, markedly elevated lactate dehydrogenase (LDH) and interleukin-10 (IL-10) provided key clues, and definitive diagnosis was achieved via a multi-site incisional RSB including subcutaneous fat. In comparison, the 10 literature-retrieved cases (*n* = 11 in total) universally demonstrated typical CES but posed significant diagnostic challenges due to the non-specificity of early symptoms and high heterogeneity on initial spinal MRI, which frequently lacked direct space-occupying evidence or appeared entirely normal.

**Conclusion:**

Clinicians should maintain a high index of suspicion for IVLBCL in patients presenting with CES when initial imaging findings fail to reflect the clinical severity, particularly when accompanied by systemic laboratory abnormalities such as elevated LDH. While serum IL-10 may serve as a potentially useful adjunctive biomarker to raise diagnostic suspicion, prompt initiation of a RSB enables early detection of this disease and minimizes diagnostic delays.

## Introduction

Intravascular large B-cell lymphoma (IVLBCL) has an incidence of only approximately 0.5 per million. Due to the unique nature of tumor cells growing within blood vessels, it often leads to “negative” or “non-specific” artifacts in imaging examinations. Clinically, IVLBCL is categorized into the classic type, the cutaneous type, and the hemophagocytic syndrome-associated type (1). Although its multi-system involvement is widely recognized, when it presents with isolated cauda equina syndrome (CES) as the initial manifestation, it is highly susceptible to being misidentified as conventional spinal degenerative changes or inflammatory radiculopathy. Previous studies have mostly focused on the systemic symptoms or central nervous system involvement of IVLBCL, whereas systematic analysis targeting the involvement of this specific anatomical site—CES—is relatively scarce. By reporting a case of IVLBCL presenting with CES and performing a literature review of 10 similar cases reported in the literature over the past 20 years, this study aims to analyze the clinical features of this rare phenotype to improve the diagnostic rate of the disease.

## Case presentation

**Figure 1:**
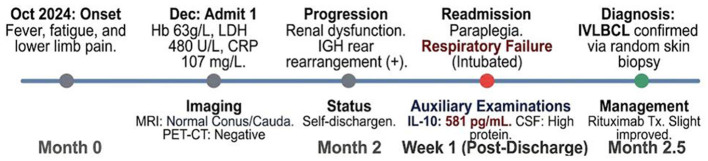
Timeline of the clinical course, illustrating the discrepancy between initial negative spinal MRI findings and subsequent rapid clinical deterioration. The definitive diagnosis of IVLBCL was confirmed via random skin biopsy, prompted by abnormal auxiliary examinations including IL-10 elevation.

A 61-year-old male presented in October 2024 (Figure 1) with generalized fatigue, intermittent low-grade fever (peak temperature <38 °C), thigh and buttock pain, numbness, and anorexia. Upon admission to the Hematology Department in December 2024, initial investigations revealed a white blood cell count of 3.94 × 10^9^/L (reference range: 3.5–9.5 × 10^9^ /L), hemoglobin of 63 g/L (130–175 g/L), and platelets of 111 × 10^9^/L (125–350 × 10^9^ /L). Biochemical analysis indicated hypoalbuminemia, with lactate dehydrogenase (LDH) at 480 IU/L (120–250 IU/L), C-reactive protein (CRP) at 107.75 mg/L (0–6 mg/L), and serum ferritin at 1,248 ng/ml (24–425 ng/ml). Abdominal ultrasonography showed no significant lymphadenopathy around the retroperitoneal great vessels. Plain MRI of the lumbar spine (Supplementary Figures 1C, D) and sacrococcygeal spine (Supplementary Figure 1E) revealed degenerative changes and disc bulging/protrusions at L2-S1, but the signals of the conus medullaris and cauda equina appeared normal. Brain MRI showed mild cerebral atrophy, bilateral mastoiditis, and sinusitis (Supplementary Figures 1A, B). Cervical MRI showed disc herniation at C3-7 and endplateitis at C5-6. Pelvic MRI revealed bilateral iliacus muscle edema but no abnormalities in the prostate or seminal vesicles. PET-CT identified small patchy shadows in the lower lungs (suggestive of infection), splenomegaly, and chronic pulmonary changes, with no evidence of highly metabolic lymphoma masses.

During hospitalization, the patient developed urinary retention and renal insufficiency, requiring catheterization and hydration therapy. Bone marrow aspiration showed anemia with erythroid hyperplasia, while IGH rearrangement was positive. Flow cytometry of the bone marrow identified a monoclonal B-lymphocyte population (0.80% of 300,000 nucleated cells) with the phenotype CD19dim+CD20+CD22+CD5-CD10-CD23-CD81-CD103-CD200-FMC-7-sKappa+sLanbda-, suggesting a B-cell proliferative disorder. The patient declined lumbar puncture, contrast-enhanced MRI, and bone marrow biopsy, subsequently requesting discharge.

**Figure 2:**
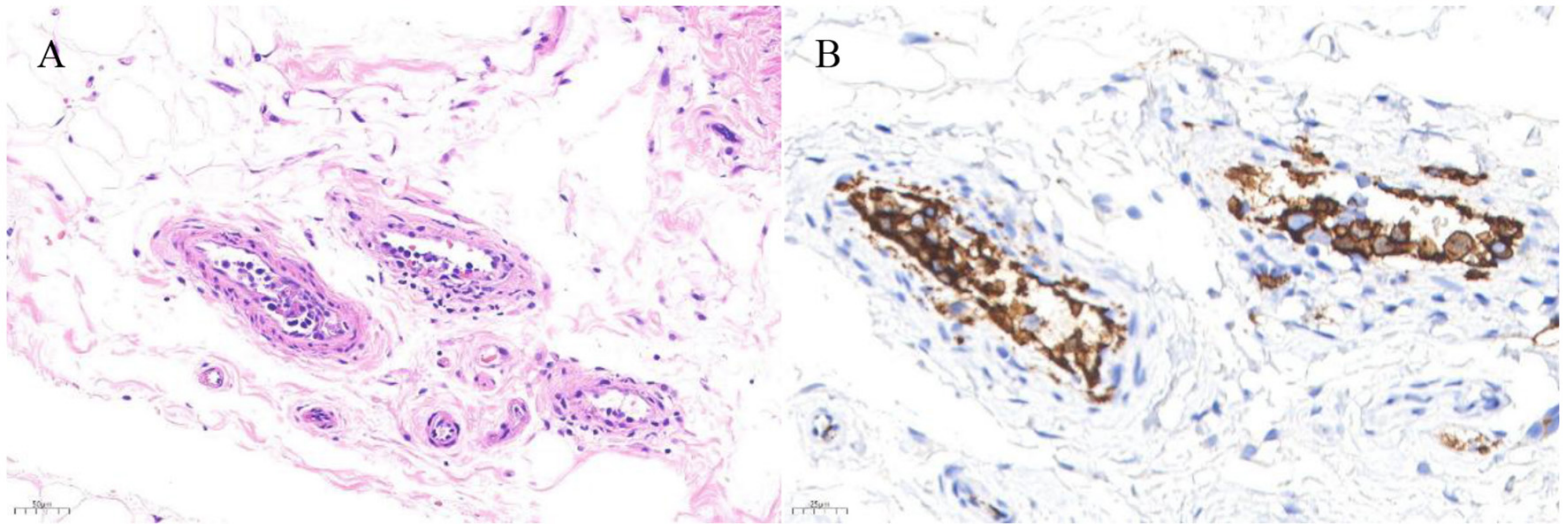
Subcutaneous fat biopsy showing small vessel involvement. HE staining (×200) **(A)**. CD20 immunohistochemical staining showing CD20-positive tumor cells (×400) **(B)**.

One week later, the patient was readmitted due to an acute exacerbation of lower limb weakness and inability to walk, rapidly progressing to respiratory failure requiring endotracheal intubation. Physical examination showed grade 2/5 muscle strength on the Medical Research Council (MRC) scale in the left lower limb and grade 1/5 in the right lower limb, with diminished tendon reflexes and hypoesthesia below the L1 level and in the perianal region. Repeat laboratory tests showed hemoglobin at 94 g/L, platelets at 59 × 10^9^/L, LDH at 381 IU/L, and CRP at 47.41 mg/L. Notably, IL-6 70.64 pg/ml (0–5.3 pg/ml) and IL-10 581.21 pg/ml (0–4.91 pg/ml) were significantly elevated. Cerebrospinal fluid (CSF) analysis revealed an elevated white blood cell count of 27 × 10^6^/L (0–8 × 10^6^/L) and an increased total protein level of 1.345 g/L (0–0.45g/L). Follow-up MRI of the entire spine and brain showed no interval changes. A random skin biopsy (RSB) of the bilateral thighs revealed several atypical lymphocytes within small blood vessels. Immunohistochemistry confirmed these cells as CD20+, Ki-67 (index ~60%), MUM1+, Bcl-6+, CD3-, and CD10-, consistent with non-Hodgkin lymphoma (Figure 2). Bone marrow biopsy later showed lymphocytosis and myeloid hyperplasia. Electromyography indicated neurogenic damage in both lower limbs. A final diagnosis of intravascular large B-cell lymphoma (IVLBCL) was established. Following one dose of Rituximab (375 mg/m^2^), the patient's lower limb weakness slightly improved, but the family subsequently elected to transition to palliative care at a local hospital. Regrettably, the patient's final survival status could not be determined, as he was lost to follow-up after the transfer.

## Discussion

Intravascular large B-cell lymphoma (IVLBCL), first described in 1959, is generally recognized as a rare and distinct clinicopathological subtype of diffuse large B-cell lymphoma (DLBCL) (2). It is pathologically characterized by neoplastic lymphocyte proliferation restricted to the vascular lumina (3). Although systemic involvement is common—frequently affecting the skin, CNS, and hematopoietic system—CES as an isolated presenting symptom remains clinically exceptional. Our pooled analysis (Table 1) reveals a significant diagnostic delay in this subtype.

**Table 1:** Summary of cases of IVLBCL with CES.

Patient	References	Age/sex	Initial presentation	^*^Diagnostic delay (months)	MRI manifestations	Diagnosis method
1	Current case	61/M	Weakness, radicular pain, numbness, and sphincter dysfunction	2.5	Normal	RSB
2	Ohno et al. (11)	46/F	Lower limb weakness, sphincter dysfunction	—	Normal	Bone marrow biopsy
3	Aznar et al. (12)	47/M	Distal paresthesia and dysesthesia of lower limbs	4	Focal fusiform thickening of conus medullaris	Autopsy
4	Piyatanont et al. (13)	77/M	Progressive numbness and weakness of lower limbs	—	Cauda equina nerve root enhancement	RSB
5	Abuzinadah et al. (14)	70/M	Perineal numbness, urinary retention	5	Diffuse cauda equina nerve root enhancement	Nerve root biopsy
6	Coulibaly et al. (15)	51/M	Lower limb weakness, sphincter dysfunction	1	T2-hyperintensity of conus; enlarged lumbar roots	Adrenal gland biopsy
7	Wada et al. (16)	62/M	Lower limb paresthesia, sphincter dysfunction	1	Cauda equina hyperintensity	RSB
8	Costanzi et al. (6)	58/F	Acute radicular pain, urinary dysfunction	1	Slight/widespread caudal root enhancement	Autopsy
9	Yu and Govindarajan (17)	55/M	Sphincter dysfunction, saddle anesthesia	—	Neuroforaminal narrowing (L5/S1)	Adrenal gland biopsy
10	Gonzalez-Pombo et al. (18)	Middle-aged/F	Lower limb weakness, saddle anesthesia, sphincter dysfunction	—	Expansive intramedullary lesion (conus)	Nerve root biopsy
11	Kitahara et al. (19)	72/M	N/R	2	T2-hyperintensity of conus medullaris	RSB

IVLBCL, intravascular large B-cell lymphoma; T2WI, T2-weighted imaging; N/R, not reported; —, unknown/not available; RSB, random skin biopsy.

^*^“Diagnostic delay” is standardized and defined as the time interval from the onset of definitive CES symptoms to definitive pathological confirmation.

CES results from lesions at the L3-L5 vertebral levels, manifesting as neurological deficits involving the L3-S5 nerve roots (4). Clinically, this syndrome presents as a distinct constellation of symptoms, including severe sphincter and urinary dysfunction, saddle anesthesia (sensory loss over the perineum, buttocks, and inner thighs), and bilateral lower limb neurogenic deficits (5). However, because IVLBCL typically does not form solid masses, spinal imaging frequently fails to provide direct evidence of a space-occupying lesion despite the presence of severe neurological deficits. Conventional imaging modalities, such as lumbar MRI, often reveal only non-specific nerve root thickening or enhancement, and some cases may even present with entirely negative findings in the early stage (6), which is consistent with the findings summarized in our literature review (Table 1).

Given the limitations of imaging, laboratory markers provide critical early warnings. While elevated LDH and β2-microglobulin are seen in approximately 90% of cases, they lack specificity (3). In our case, a highly elevated serum interleukin-10 (IL-10) level was detected. Accumulating data indicate that serum IL-10 may serve as a promising adjunctive biomarker to assist in the differential diagnosis of IVLBCL. For instance, previous research reported that a threshold of >95.65 pg/ml yielded 80% sensitivity and 100% specificity for IVLBCL within the studied cohort (7). Therefore, the elevated IL-10 in our patient served as a auxiliary clue that prompted the subsequent random skin biopsy for definitive diagnosis.

The diagnosis of IVLBCL primarily relies on histopathology. A multi-site incisional RSB including subcutaneous fat offers a safer alternative with a reported 77.8% sensitivity (8). Crucially, malignant cells preferentially sequester in the microvasculature of subcutaneous adipose tissue. Therefore, it is recommended that clinicians consider performing an appropriately deep RSB in fat-rich anatomical sites (such as the abdominal wall or medial thighs) to ensure that adequate subcutaneous adipose tissue is sampled, even in the absence of macroscopic skin lesions (9).

Although the traditional CHOP regimen (comprising cyclophosphamide, doxorubicin, vincristine, and prednisone) has been a standard approach, its efficacy remains limited. Previous studies have demonstrated that the integration of Rituximab with the CHOP regimen (R-CHOP) significantly improves both overall survival (OS) and progression-free survival (PFS) (10). Nevertheless, managing IVLBCL continues to present formidable challenges. Advances in immunotherapy and targeted agents have introduced novel therapeutic avenues.

## Conclusion

In patients exhibiting acute or subacute CES without definitive evidence of a space-occupying lesion on imaging, clinicians should maintain a high index of suspicion for IVLBCL, particularly when accompanied by auxiliary laboratory abnormalities such as elevated LDH and IL-10 levels. Early implementation of RSB is instrumental in shortening the diagnostic delay, facilitating the timely initiation of therapy, and ultimately improving patient outcomes.

## Data Availability

The original contributions presented in the study are included in the article/Supplementary material, further inquiries can be directed to the corresponding authors.
