# Supplementary material for: Case Report: Intravascular large B-cell lymphoma presenting with cauda equina syndrome: a rare case and diagnostic dilemma
Source: Front Med (Lausanne). 2026 Aug 5;13:1873276. doi: 10.3389/fmed.2026.1873276 (PMC13485540; doi:10.3389/fmed.2026.1873276)
Supplement: Supplementary file 1 [file Image_1.pdf]

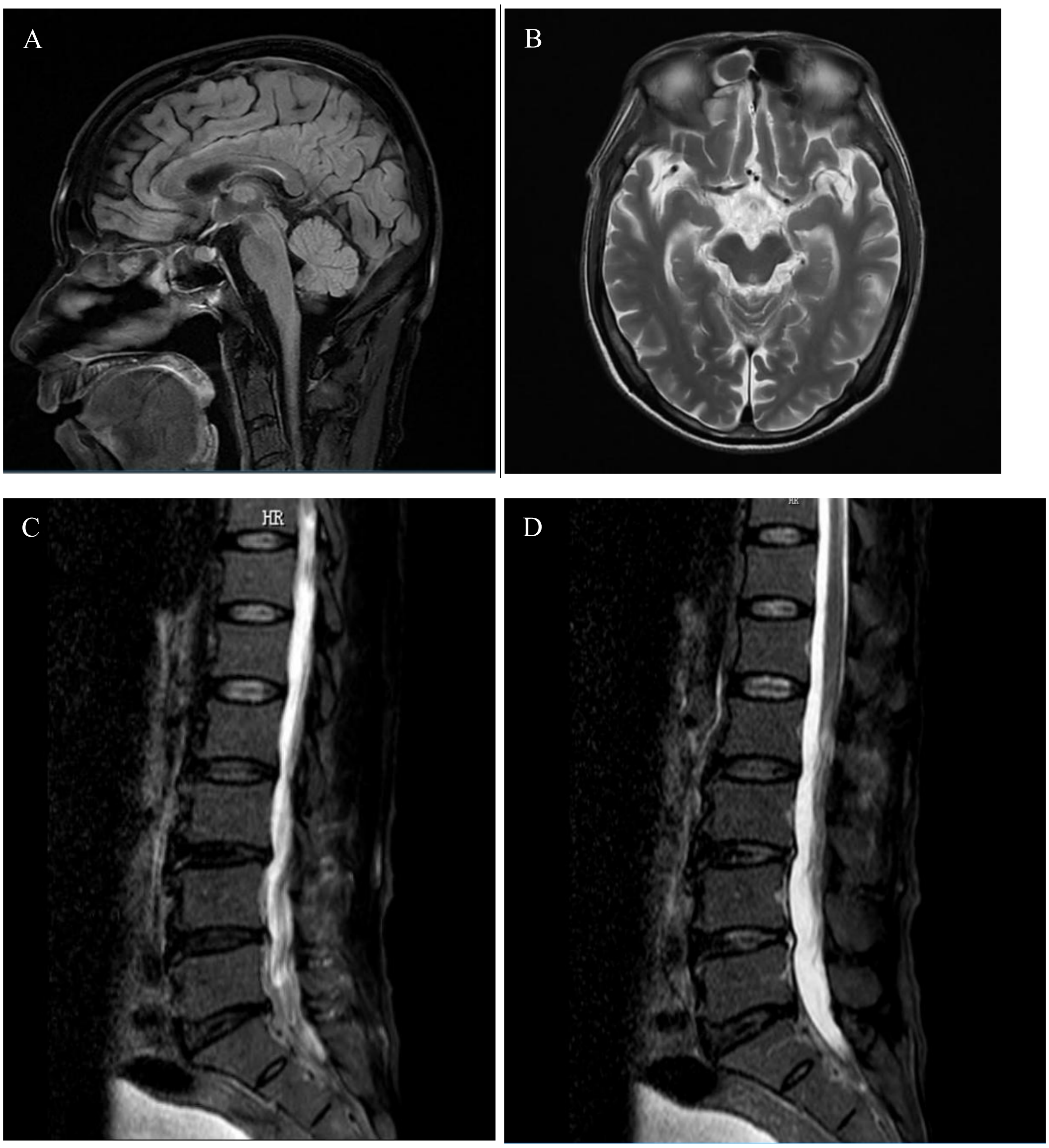

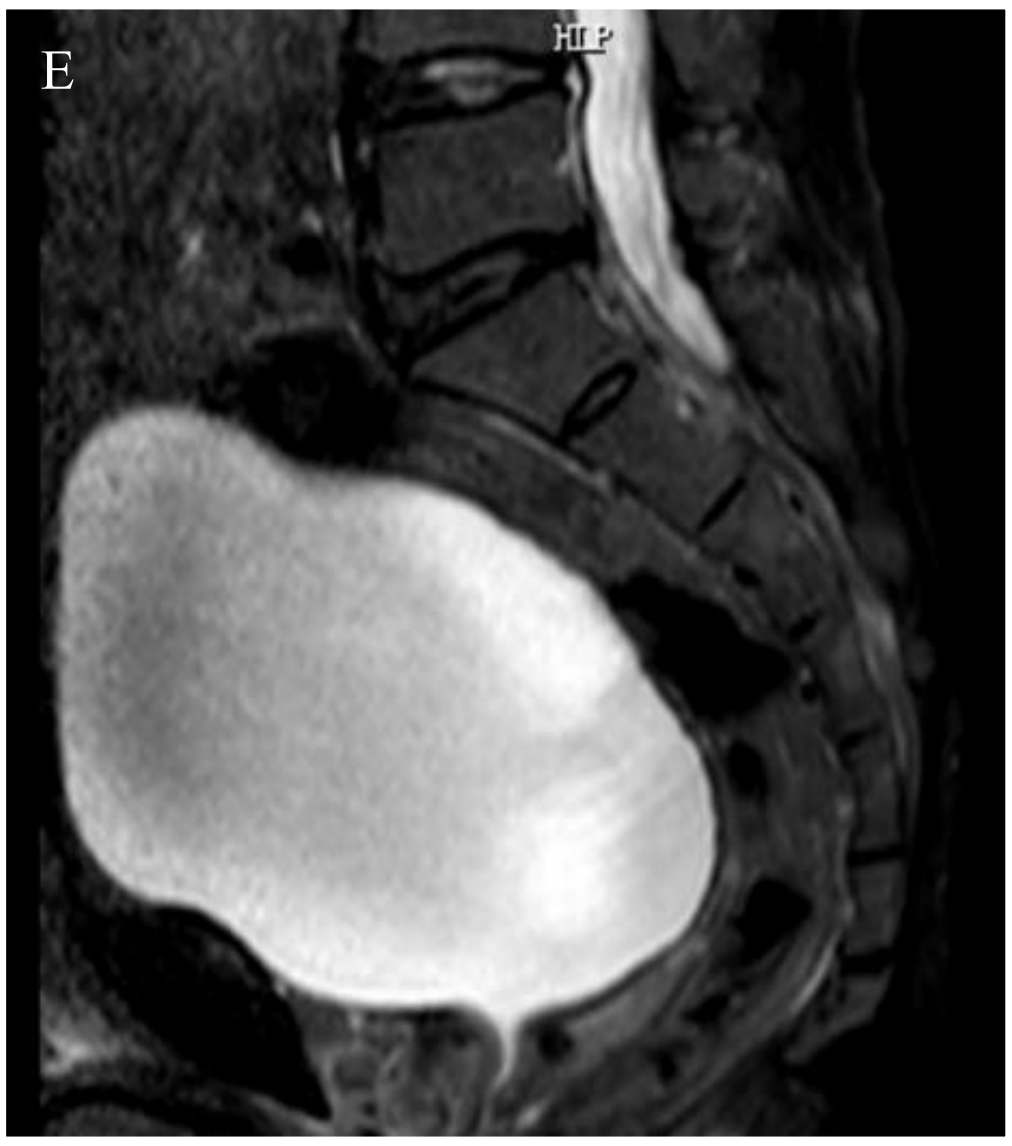

Supplementary Fig 1. Neuroradiological evaluation of the patient. (A, B) Brain MRI: Sagittal T2-FLAIR **(A)** and axial T2-ARMS **(B)** images showing normal brain structures without abnormal signals or occupying lesions in the brain parenchyma, brainstem, or cerebellum. Mild cerebral atrophy, paranasal sinusitis, and mastoiditis are present. **(C, D)** Lumbar spine MRI: Sagittal T2W-TSE **(C)** and sagittal T2W-SPAIR **(D)** images showing typical lumbar degeneration. Although mild disc bulging at L2-4 and protrusion at L4-S1 with slight compression of the thecal sac are present, the spinal cord conus and cauda equina show no abnormal signal intensities. **(E)** Sacrococcygeal spine MRI: Sagittal T2W-SPAIR image showing typical sacrococcygeal degeneration with mild osteophyte formation, with no abnormal signals or occupying lesions within the sacral canal.
